# Relevance of the Ejaculate Fraction and Dilution Method on Boar Sperm Quality during Processing and Conservation of Seminal Doses

**DOI:** 10.3390/vetsci8120292

**Published:** 2021-11-27

**Authors:** Blanca Sebastián-Abad, Pedro José Llamas-López, Francisco Alberto García-Vázquez

**Affiliations:** 1 Cmno De Los Clementes Sn, GTC Spermatica Reproducción, 30817 Lorca, Spain; blanca.s.a@um.es (B.S.-A.); pedrojosellamas@outlook.es (P.J.L.-L.); 2 Departamento de Fisiología, Facultad de Veterinaria, Campus Mare Nostrum, Universidad de Murcia, 30100 Murcia, Spain

**Keywords:** porcine, preservation, sperm function, semen agitation, storage

## Abstract

During boar semen processing and distribution, maximizing the work protocols in the laboratories becomes essential for the conservation of seminal doses. One of the recent implementations in the boar studs to improve efficiency has been semi-automatic semen collection systems, which do not allow to discard fractions of the ejaculate. The objective of this work was to evaluate the dilution method and vibrations (simulating delivery transport) effect on sperm quality (motility, viability, morphology, thermo-resistance test) according to the fraction of ejaculate collected. Two different fractions of the ejaculate were obtained [rich fraction (RF); total fractions (TF)] from six boars, and two dilution methods applied [pouring the extender over the semen (control; ES); pouring the semen over the extender (reverse; SE)]. The seminal doses (2000 × 10^6^ sperm/50 mL) were preserved for 5 days. The results showed that the fraction collected affects sperm quality (better total and progressive motility, and faster sperm in TF; *p* < 0.05) regardless of the dilution method applied. However, these differences diminished after submitting the semen to the thermo-resistance test, with only differences in sperm viability being observed (*p* < 0.05). When seminal doses were subjected to vibrations, the sperm viability was more affected in the TF than in the RF group (*p* < 0.05). In conclusion, using the TF ejaculate leads to comparable results to the RF in sperm quality during storage regardless of the dilution method applied. However, the vibrations of seminal doses are more affected in doses prepared with TF than with RF, although more factors should be included to approach the real conditions during transport.

## 1. Introduction

Over the last decades, artificial insemination (AI) has been increasingly improving in the swine industry [1]. With the use of new tools and technologies, both the volume and the number of spermatozoa per dose of insemination have decreased without impairing fertility results [1,2,3]. However, these results would not be possible without the appropriate key measures in quality control of semen, which are essential to extend sperm life and its fertilizing capability [4]. Therefore, the industry is continually challenging for new procedures—e.g., semen processing—trying to optimize the efficiency of the swine industry in terms of reproduction [1].

Semen is routinely collected from boar studs—intervals of 5–7 days between each boar extraction [5]—for further processing in the laboratory and distribution. The ejaculate of the boar is composed of spermatozoa and seminal plasma (SP) that comes from the epididymis and accessory sexual glands. The entire ejaculate consists of three different consecutive fractions [6,7]. The first one is the pre-sperm fraction which is composed of urine and smegma coming from the urethra and bulbourethral glands mostly and presents a watery aspect. The second part of the ejaculate is the sperm-rich fraction (RF), containing most of the spermatozoa of the ejaculate, provided by the testis and epididymis. They are diluted in SP from the vesicular glands and the prostate. This fraction is characterized by its dense white color and is always collected [8]. Then, the post-sperm rich fraction (PSRF), with quite a smaller number of spermatozoa and high content in SP from the accessory glands. It can be distinguished by its watery aspect [6]. Lastly, the bulbourethral glands deliver the tapioca, which is always discarded during ejaculate collection, that coagulates in contact with SP to maintain the ejaculate in the female reproductive tract during natural mating [9]. The first fraction of the ejaculate is always discarded [8], however, the inclusion of the third fraction is certainly controversial [10] and depends also on which collection method is used. Classically, the “gloved hand method” [11] has been used for decades for collecting the RF of the ejaculate and discarding the PSRF, which remains as the method of choice [12]. More recently, because of the technical improvement of studs, a semi-automated technology for semen collection has been created [13], which was already a routine method for extraction in some countries such as Holland [14]. This semi-automated method consists of an artificial vagina controlled by air pressure by a handle in which the penis is gripped. This method is less time-consuming and more efficient [15] than the manual method; however, the PSRF cannot be discarded, being the bulk ejaculate—RF and PSRF—collected [16]. There is no current consensus about the inclusion of PSRF for preparing the seminal doses because it must be pointed out that different quantities of SP from different ejaculate fractions can affect sperm quality and semen conservation [17,18,19,20,21]. For this reason, there is a lack of studies showing the comparative effect of RF only and the bulk ejaculate.

Following the collection, the semen is diluted on a commercial extender to obtain an adequate concentration for preparing AI seminal doses and to be refrigerated—15–17 °C; [22]—for several days—3–5 days—in optimal conditions. However, the dilution step is a critical point because spermatozoa can undergo loss of motility and membrane integrity due to the so-called “dilution effect” [8,23]. This effect depends on some factors such as temperature [24], dilution technique [25], or the seminal plasma [26]. Then, there are different semen dilution protocols used in the boar studs. On one hand, semen dilution could be performed in one or two steps [27]. The one-step dilution consists in mixing the semen and the extender isothermally within the first 30 min after collection [22]; and the two-step procedure requires a previous dilution in a ratio of 1:1 or 1:2, and a subsequent isothermal or hypothermal final dilution [24,28]. On the other hand, during the dilution process, the extender could be poured over the semen—“control method” [29]—or the “reverse method”—semen over the extender. The last two methods have proved to give comparable results in semen quality using the same fraction of the ejaculate, although the control method causes the formation of foam, which may entail hygienic risks [25]. However, the effect of the dilution method in different ejaculate fractions has not been tested, and it must be pointed out that the different quantities of SP could influence sperm quality as well [26]. Since this fluid contains more proteins, specifically albumin [30,31], the bulk ejaculate may cause more foam during dilution depending on the applied method.

Once semen is diluted, the doses are packaged, tempered [32], and stored in refrigerators at 15–17 °C. The centralization in the production of boar semen doses leads to a requirement for transportation and the optimization of the conservation of the quality during this process [27]. However, only a few investigations have been performed evaluating the effect of transport on the seminal doses [33,34].

Considering the actual tendency to collect the bulk ejaculate, the different repercussions on the quality of liquid semen depending on the fraction collected—RF against the bulk ejaculate-TF—should be investigated. The different fractions of the boar ejaculate differ in composition which could affect the sperm quality during the seminal dose preparation—e.g., dilution method—conservation or distribution—e.g., vibrations during transport. Therefore, the objectives of this study were: (1) To evaluate the impact of the different fractions collected of the ejaculate—RF vs. TF—, and the dilution method applied —“control” vs. “reverse”—on the sperm quality during conservation—up to 5 days; (2) To analyze the effect of a simulated transport —vibrations— of the seminal doses on the sperm quality depending on the dilution method—“control” vs. “reverse”—and ejaculate fractions—RF vs. TF—used.

## 2. Materials and Methods

### 2.1. Animals

The ejaculates were obtained from 6 fertility proved boars (Duroc Axiom; 15.4 ± 1.4 months) for AI purposes from a commercial boar stud [CTG Spermatica Reproduccion (Lorca, Spain)], and the seminal doses were used for the study. During the trial, boars were housed in individual pens—according to the European Commission Directive for Pig Welfare—with grating or sawdust. Their nourishment consisted of 3 kg per day of swine feed, based on barley, corn, and wheat, and water available ad libitum. The boar stud had controlled conditions of humidity (50%) temperature (24 °C) and light (16 h light/8 h darkness; 307 pro, BigDutchman^®^, Vechta, Germany).

### 2.2. Semen Collection and Seminal Doses Preparation

A total of two seminal extractions per boar (*n* = 12 ejaculates; Table 1) were performed with an interval between them of 5–7 days—~1.25 services per week. The “gloved-hand” method was used for semen collection using a Thermal semen collection flask (Tecno-Vet S.L., Barcelona, Spain) previously tempered at 40 °C, and no extender was placed on it. The pre-sperm phase of the ejaculate was discarded, and the gel fraction of the semen was removed using a filter (Tecno-Vet S.L., Barcelona, Spain). For each extraction, different fractions of the ejaculate were collected—distinguished by sight—: (1) RF (rich fraction): composed by the sperm rich fraction of the ejaculate, formed by spermatozoa mostly [6] which is characterized by a dense white color. The collection in this case ended with the transition to water-like liquid, which entails the beginning of the PSRF; (2) TF (total fractions): including the RF and PSRF. The mean (±SD) volumes obtained were 99 ± 64.7 mL for RF and 244.3 ± 92.3 mL for TF.

**Figure 1:**
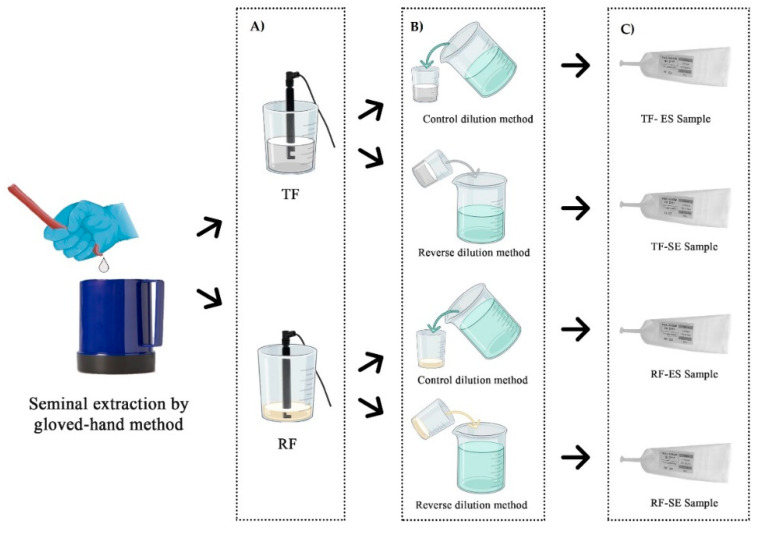
The semen collection by the gloved-hand method is shown and the steps during the preparation of the seminal dose are the following: (**A**) measurement of the sperm concentration by Metrosperm (Tecno-Vet S.L., Barcelona, Spain) in RF (rich fraction) and TF (total fractions) ejaculates; (**B**) application of the two different dilution methods (control: pouring the extender over the semen-ES; reverse: pouring the semen over the extender-SE; (**C**) packaging of seminal doses from the different experimental groups. Figure created in Biorender.com, accessed on 21 October 2021.

The inclusion criteria of the ejaculates were: a minimum of 70% of progressive motile sperm and 75% of normal spermatozoa. Once collected, the spermatozoa concentration was evaluated through the MetroSperm (Tecno-Vet S.L., Barcelona, Spain), and the extender (270–340 mOsm/kg; pH: 6.7–7.4; Aprocell Plus, IMV Technologies, L’Aigle, France) was added at 32 °C to obtain a similar concentration in every sample (39.8 ± 3.7 × 10^6^ spermatozoa/mL). During this process, the ejaculate—RF or TF—was divided into two parts to perform the different dilution methods. The control dilution method was performed by pouring the extender over the semen (ES; [29]); and the reverse method pouring the semen over the extender (SE). The seminal doses of each boar (50 mL) were packaged in AI tubes (Tecno-Vet S.L., Barcelona, Spain). Immediately after filling, seminal doses were kept at a controlled room temperature (20–22 °C) until stabilization [32] and refrigerated at 15 °C for further analysis. A summary of the preparation for semen collection and seminal doses is shown in Figure 1.

### 2.3. Assessment of Sperm Motility

The computer-assisted sperm analyses (CASA system, Microptic S.L., Barcelona, Spain) was used to assess the motility of the sperm. The computer was connected to a phase-contrast microscope (10×; Nikon Eclipse E200, Tecno-Vet S.L., Barcelona, Spain). Samples were evaluated in pre-warmed disposable chamber slides (Goldcyto Slides, Microptic S.L, Barcelona, Spain) using 25 μL per sample. During analyses, 3–5 fields were recorded including at least 1000 spermatozoa in total. CASA setting parameters included particles size area from 10 to 80 mm^2^ and 25 frames per second. Their trajectories were analyzed according to the following classification: static (<10 mm/s), slow (10–25 mm/s), intermediate (>25–50 mm/s) and rapid (>50 mm/s) spermatozoa. The following parameters were analyzed: progressive motility (%) and total motility (%) including slow (%), intermediate (%), and rapid (%) spermatozoa.

### 2.4. Assessment of Sperm Morphology

The spermatozoa morphology was evaluated by light microscopy (40× objective; *n*-180 M binocular microscope, Tecno-Vet S.A., Barcelona, Spain) previously immobilized with diluted formaldehyde (1%). Ten microliters of semen sample were placed in a slide and covered with a coverslip, and the following abnormal forms [35] were recorded by percentage from a total of 100 spermatozoa: normal, proximal cytoplasmic droplet, distal cytoplasmic droplet, folded tail, coiled tail, head defects, and midpiece defects.

### 2.5. Assessment of Sperm Viability (Plasma Membrane Integrity)

The viability was evaluated through eosin/nigrosine staining. One droplet of staining and an equal volume of the semen sample were mixed and smeared on a slide. Then, a total of 100 spermatozoa per sample were counted under light microscopy (40× objective; *n*-180 M binocular microscope, Tecno-Vet S.L., Barcelona, Spain) and were classified according to their membrane integrity into dead (rose-colored spermatozoa) or alive (colorless spermatozoa [36]).

### 2.6. Simulation of Vibration Emissions

To simulate vibration emissions during shipping and delivery of boar seminal doses, they were submitted to vibration by circular horizontal frequencies and a rotation of 300 rpm (IKA Vibrax VXR Basic S1^®^, Staufen, Germany). For that, seminal doses were kept in the dark and vibrated for 6 h (an estimation of transport time of seminal doses based on the experience in the boar stud of the study) at 15 °C of temperature [34]. The doses were kept at 15 °C until assessment.

### 2.7. Thermo-Resistance Test

The thermo-resistance test simulates the time that the spermatozoa spend in the female genital tract by exposing it to 38 °C for a long time [28]. For that purpose, an aliquot of 15 mL from seminal doses was submitted to a constant temperature of 38.0 ± 0.5 °C in a water bath for 5 h. Following the incubation, motility, viability, and morphology of the spermatozoa were assessed (as previously described) to test their thermo-resistance.

### 2.8. Experimental Design

The present study was divided into two different experiments.

#### 2.8.1. Experiment 1: Assessment of Dilution Method and/or Ejaculate Fraction on Spermatozoa Parameters during 5 Days of Seminal Doses Refrigeration

**Figure 2:**
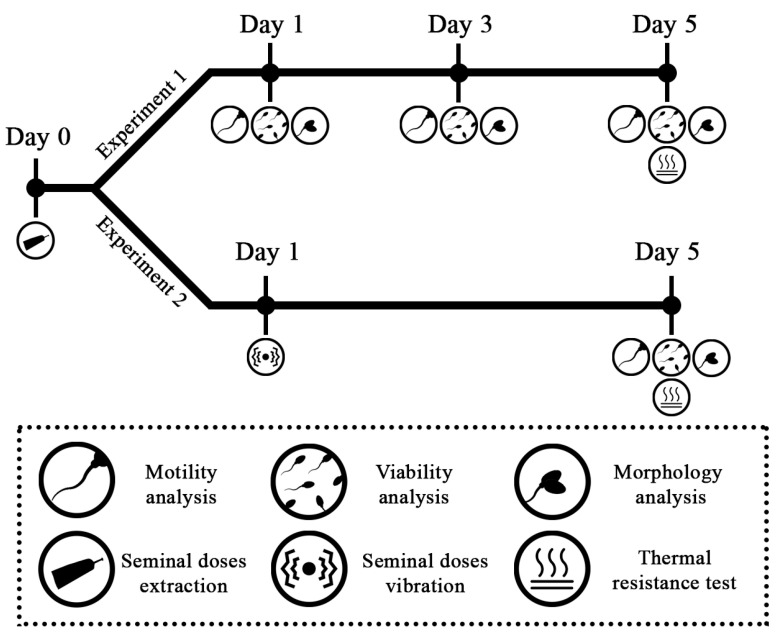
The experimental design of the Experiment 1 and Experiment 2 is represented. The different assessments on sperm quality performed in specific days during conservation (days 1, 3, and 5), and the two tests performed (thermo-resistance test and seminal doses vibration) are indicated according to the symbols below.

To test the effect of the fraction and dilution method on sperm quality of the doses, two separated ejaculates (RF and TF) from the six boars that were also differently diluted (ES and SE dilution methods) were studied, which meant four experimental groups per boar (Table 1). Sperm quality—motility, viability, and morphology—were evaluated on days 1, 3, and 5 of conservation. In addition, every dose was submitted to the thermo-resistance test on day 5 of conservation and subsequently assessed. A summary of the experimental design is shown in Figure 2.

#### 2.8.2. Experiment 2: Effect of Seminal Dose Vibration on Sperm Quality Depending on the Ejaculate Fraction Collected and/or Dilution Method Used

To assess the effect of the ejaculate fraction and dilution method on sperm quality after transport, a seminal dose of each fraction collected (RF and TF), dilution method applied (SE and ES), and boar (*n* = 6) used as described (Table 1) was vibrated on day 1 after extraction (simulating the day it would be transported). Subsequently, on day 5 of conservation (maximum day of conservation at the farm [34]), the effect on sperm quality was analyzed through sperm motility, viability, and morphology. Additionally, the thermo-resistance test was performed on day 5 of conservation and subsequently analyzed. In this manner, the effect suffered by vibration (day 1) on the doses was evaluated on the limit day of use for insemination at farms (day 5). A summary of the experimental design is shown in Figure 2.

### 2.9. Statistical Analysis

Data were analyzed using the IBM SPSS 24 Statistics package (SPSS, Chicago, IL, USA) and Statistic Analysis Software (SAS, University Edition 2016). The assumption of normality was checked by the Shapiro–Wilk test. The data for experiment 1 (seminal doses conservation) were analyzed by ANOVA with repeated measurements (PROC MIXED). The model included the experimental groups (RF-ES, RF-SE, TF-ES, TF-SE), the time related to experimental groups (1, 3, and 5 days), and their interaction as the main effect. For multiple pairwise comparisons (thermo-resistance test of experiment 1 and experiment 2), data were analyzed by ANOVA with post hoc Tukey test. Data that were not normally distributed were analyzed by the Kruskal–Wallis test. When only the effect of seminal doses vibration (vibration vs. non-vibration, experiment 2) was analyzed, the Student’s *t*-test (progressive motility, sperm velocities, morphology, and viability) and U-Mann Whitney test (total motility and viability) were applied. Differences were considered statistically significant at *p* < 0.05. Data are expressed as the mean ± SEM (standard error of mean).

## 3. Results

### 3.1. Experiment 1: Assessment of Dilution Method and/or Ejaculate Fraction on Spermatozoa Parameters during 5 Days of Seminal Doses Refrigeration

The results are shown in Table 2 and the Supplementary File. The total motility was significantly higher in the TF-ES group compared with RF groups (ES and SE; *p* < 0.05) without differences between them. No differences were observed when groups using the same fraction of the ejaculate (RF or TF), but different dilution methods (ES or SE), were compared. Moreover, the percentages of rapid sperm and progressive motility were higher (*p* < 0.05) in the seminal doses prepared with TF than in RF, independently of the dilution method. In this sense, the percentages of slow sperm and non-progressive motility were higher (*p* < 0.05) in the seminal doses prepared with RF than TF, independently of the dilution method. No differences were found in viability and normal morphology between the experimental groups. Any of the parameters showed an interaction between time and groups.

However, the differences in sperm motility between groups were scattered after 5 days of seminal doses storage and subsequent thermic incubation (thermo-resistance test) for 300 min at 38 °C (Table 3), although significant differences in viability (*p* < 0.05) were observed. Higher sperm viability was found in the RF-SE group compared to TF-SE (*p* = 0.033), but both were similar to the other groups (RF-ES and TF-ES).

### 3.2. Experiment 2: Effect of Seminal Dose Vibration on Sperm Quality Depending on the Ejaculate Fraction Collected and/or Dilution Method Used

The total sperm motility and viability were reduced by the vibration of the seminal doses independently of the experimental group (83.9 ± 1.6 vs. 89.1 ± 1.4%, *p* = 0.021; and 91.4 ± 0.7 vs. 95.5 ± 0.4%, *p* < 0.001, respectively). The rest of the parameters studied were not affected by the vibrations. When the effect of vibration over the seminal doses was analyzed comparing all the experimental groups (Table 4), only significant differences were observed in viability (*p* < 0.05). The vibrated samples presented a similar percentage of sperm viability between them (*p* > 0.05) independently of the dilution method and fraction collected. Moreover, non-vibrated doses showed similar values as well (*p* > 0.05). The highest values of sperm viability corresponded to TF-SE and RF-ES non-vibrated doses but were statistically similar to the rest of the groups except TF-ES and TF-SE vibrated doses (*p* < 0.05) which showed lower values. No differences were found in total sperm motility, velocity, progressive motility, or morphology between any of the experimental groups. In addition, when sperm quality was compared between vibrated and non-vibrated doses from the same experimental group, no differences were observed for any parameter but for viability. The vibrated doses from groups TF-ES and TF-SE significantly differed in sperm viability compared with non-vibrated doses within the same experimental group (*p* < 0.05 and *p* < 0.01 respectively) which showed lower values.

In the case of doses submitted to the thermo-resistance test, the only significant differences observed between experimental groups (Table 5) were sperm viability (*p* < 0.05). The vibrated doses were equally affected by the heat, with similar values between them (*p* > 0.05), as with those non-vibrated doses (*p* > 0.05) independently of both, fraction, and dilution method. After the test, the highest value of sperm viability was observed in non-vibrated doses from group RF-SE, which differed compared to vibrated doses from TF-SE and TF-ES (*p* = 0.015). The parameters of motility, velocity, progressive motility, and morphology remained without significant differences between groups (*p* > 0.05). Additionally, only viability showed differences when comparing vibrated and non-vibrated doses from the same experimental group, specifically, RF-SE (*p* < 0.05).

## 4. Discussion

Currently, boar studs are essential in the swine industry due to their role in the frame of porcine AI. The optimization in the production of the seminal doses is directly related to the processing of the semen [27], so it is a continuous challenge for the swine industry to improve the methodology of semen collection, manipulation, conservation, and distribution. According to the present study, the ejaculate fraction collected for seminal dose preparation influences some sperm quality parameters. In addition, the seminal doses vibration, which simulates their transport to the farms, affects the sperm viability and is differently affected according to the dilution method and the ejaculate fraction used.

Boar sperm is sensitive to dilution during semen processing [8,23]. However, the impact of this factor when different ejaculate fractions are used has not been yet elucidated. In general terms, the different methodologies of dilution and fractions of the ejaculate used in the study present optimal results after 5 days of conservation. Our results showed that the dilution method tested (SE vs. ES) does not influence sperm quality independently of the ejaculation fraction used (RF vs. TF). This fact agrees with a previous report [25], contrary to the long-established recommendation that the extender should be added to the semen. These discrepancies could be due to the final dilution rate. In the study reported by Schulze et al. [25] and in our case, the dilution rates were not high, being the concentration of sperm from 23 to 40 × 10^6^ sperm/mL, which can reduce the impact on sperm compared with higher dilution rates [37,38]. The findings here reported match with the possibility of implementing different variants of semen processing depending on the workflow of each boar stud [13,14]. The risk of mistakes during boar semen processing increases as boar studs become large (reviewed by [27]), so the importance of being able to use different protocols with very similar efficiency assures reducing discards of genetic material and consequently saving costs when some errors are performed during semen processing. Moreover, the possibility of using the reverse dilution technique (SE), independently of the ejaculate fractions used, could have several advantages [27]. For example, the reduction in foam formation which facilitates better sealing of semen during packaging and avoids the contact between the filling nozzle and the semen, is a more hygienic method. Although not measured in our study, the time using this dilution method was less compared to the control dilution (extender added to semen).

The differences observed between ejaculate fractions show better motility in TF than in the RF group, meaning more desirable conservation of sperm, including the post-sperm-rich fraction. However, other studies have demonstrated the favorable use of the sperm-rich fraction over the post-sperm-rich fraction [6,19]. This discrepancy may be related to the difference found in the composition of the SP between different swine breeds [39]. Moreover, proteomics concerned with sperm motility differs as well in different breeds [40]. Thus, the sperm of the Duroc breed used in this study could present these characteristics in sperm quality and conservation, and it would be profitable to study the effect of the fraction collected on sperm conservation and quality in different swine breeds. Besides, studies previously performed on this subject matter [6,19] did not analyze accumulative fractions as in the present study, and there could be a beneficial interaction between these fractions in the complete ejaculate.

When the thermo-resistance test was applied to the seminal doses after 5 days of conservation, the motility differences previously found between the experimental groups were mitigated. However, significant differences were found between RF and TF from ejaculates diluted by the reverse method, the TF group showing lower viability. The cells have natural defensive mechanisms to prevent the negative effects that heat stress exerts on sperm such as heat shock proteins (HSPs [41]). It has been shown that one protein of this family (heat shock cognate 71 kDa) is differentially expressed when compared between boar ejaculate fractions [30]. This fact may be related to the differences found in sperm viability between experimental groups after being submitted to the thermal stress. The co-incubation of fresh sperm with HSP8 significantly enhanced sperm viability [42]. Moreover, HSP90 maintains boar sperm motility and mitochondrial membrane potential during heat stress [43].

With the centralization of semen processing in the boar stud, the transport of the final product, the seminal doses, is starting to play a key role in assuring high quality at the reception farm [34,44]. Previously, some studies have demonstrated the deleterious effect of vibration over seminal doses [33,34] although the impact of vibration has been less pronounced in our results including the thermo-resistance test. These differences observed could be attributable to the used extender, the semen dose size, or even male genetics (as explained before). In fact, differences have been observed in sperm quality after vibration when seminal doses were prepared with different extenders [33]. The size of seminal doses used in the study was 50 mL, while the other investigations used 85–90 mL. However, the impact of this difference in size requires future investigations. Moreover, our results showed that viability was affected in those vibrated samples that contained TF, in both dilution methods, but not in RF samples. Considering these results, there seems to be evidence that the composition of the SP of the PSRF or the higher proportion of this fluid in the TF seminal doses compared with RF, influences the sperm viability during the vibration process. As mentioned, it has been shown that the protein composition of the seminal plasma differed between boar ejaculate fractions [30,31,45], and it could be one of the reasons for the differences observed. Most of these proteins belong to the spermadhesin family, and depending on the type, they can be detrimental or beneficial to sperm viability [26]. Furthermore, this different concentration could depend on the fraction of the ejaculate [46]. Besides the difference in proteomics between different fractions of the ejaculate [30], a difference in metabolomics is also present [47]. Moreover, the antioxidant capacity of the SP is lower in TF than in RF [48] which implies less protection against oxidative stress, and therefore, less sperm survival. Although the consequences of all these differences are still unknown, it is tempting to assure that they are related to the decrease in viability in TF. It is also possible that the greater amount of SP and difference in proteins could cause more foam during vibrations, as described previously [34], thus impairing viability. However, we must consider some of the limitations of this study because not all the variables during the transport have been considered such as acceleration and deceleration during the trip, type of vehicle, or the type of road surface among others [34].

## 5. Conclusions

Although the ejaculate fraction included in the seminal dose and the dilution method applied during semen processing impacted some sperm parameters, the quality of the seminal doses presents optimal results. Thus, the application of different processing methods for seminal doses must be adapted to the workflow of the boar studs to maximize its efficiency. Due to the centralization of the distribution of the seminal doses, the factors involved in transportation require special attention, since the present study has proven the negative impact of the vibration on sperm quality.

## Figures and Tables

**Table 1:** Experimental groups obtained from each boar. The fraction of the ejaculate collected (RF or TF) and the dilution method applied (SE or ES) are indicated.

Number of Boars	RF + ES	RF + SE	TF + ES	TF + SE
Boar 1	1-RF-ES	1-RF-SE	1-TF-ES	1-TF-SE
Boar 2	2-RF-ES	2-RF-SE	2-TF-ES	2-TF-SE
Boar 3	3-RF-ES	3-RF-SE	3-TF-ES	3-TF-SE
Boar 4	4-RF-ES	4-RF-SE	4-TF-ES	4-TF-SE
Boar 5	5-RF-ES	5-RF-SE	5-TF-ES	5-TF-SE
Boar 6	6-RF-ES	6-RF-SE	6-TF-ES	6-TF-SE

ES = control dilution method, extender over the semen; SE = reverse dilution method, semen over the extender. RF = sperm rich fraction; TF = the total fractions of the ejaculate.

**Table 2:** Effect of dilution method and/or ejaculate fraction on boar spermatozoa during 5 days of refrigeration (15 °C). Data include the statistical analysis of repeated measures (day 1, 3, and 5). Data are shown as the mean ± SEM.

	RF		TF	
Parameter	ES	SE	ES	SE
Total Motility (%)	88.6 ± 1.2 ^a^	89.8 ± 1.2 ^ab^	93.2 ± 1.3 ^c^	91.8 ± 1.2 ^bc^
*Rapid (%)*	51.9 ± 5.1 ^a^	53.4 ± 5.1 ^a^	61.0 ± 5.2 ^b^	58.8 ± 5.1 ^b^
*Intermediate (%)*	19.3 ± 3.3	19.3 ± 3.3	20.7 ± 3.3	19.4 ± 3.2
*Slow (%)*	17.3 ± 1.6 ^a^	16.9 ± 1.6 ^a^	11.5 ± 1.6 ^b^	13.4 ± 1.6 ^b^
Progressive Motility (%)	61.2 ± 3.8 ^a^	62.4 ± 3.8 ^a^	72.6 ± 3.9 ^b^	69.6 ± 3.8 ^b^
Non-Progressive Motility (%)	27.4 ± 3.0 ^a^	27.4 ± 3.0 ^a^	20.6 ± 3.0 ^b^	22.1 ± 3.0 ^b^
Normal Morphology (%)	93.4.8 ± 0.7	93.2 ± 0.7	93.9 ± 0.4	94.3 ± 0.5
Viability (%)	96.5 ± 0.5	96.1 ± 0.5	95.8 ± 0.5	95.8 ± 0.4

The parameters of velocity in italic (rapid, slow, and intermediate) refer to total sperm motility. ES = control dilution method, extender to semen; SE = reverse dilution method, semen to extender; RF = sperm rich fraction; TF = total fractions. Different letters (a,b,c) within a row indicate significant differences between experimental groups (*p* < 0.05).

**Table 3:** Effect of dilution method and/or ejaculate fraction on thermo-resistance of boar spermatozoa after 5 days of refrigeration (15 °C). Data are shown as the mean ± SEM.

	RF		TF	
Parameter	ES	SE	ES	SE
Total Motility (%)	37.9 ± 6.6	41.1 ± 6.3	41.5 ± 4.8	40.3 ± 3.0
*Rapid (%)*	12.0 ± 3.0	14.2 ± 3.5	14.5 ± 2.0	15.3 ± 2.2
*Intermediate (%)*	7.2 ± 1.6	8.5 ± 1.4	8.0 ± 0.9	7.1 ± 1.0
*Slow (%)*	18.6 ± 2.8	18.3 ± 2.1	18.9 ± 3.4	17.8 ± 1.2
Progressive Motility (%)	16.2 ± 3.7	19.1 ± 3.9	19.7 ± 2.0	19.4 ± 2.1
Non-Progressive Motility (%)	21.6 ± 3.3	21.9 ± 2.5	21.7 ± 3.4	20.8 ± 1.6
Normal Morphology (%)	94.1 ± 0.7	95.0 ± 0.5	94.1 ± 0.7	93.3 ± 0.7
Viability (%)	92.6 ± 1.2 ^ab^	94.3 ± 1.0 ^a^	90.0 ± 1.5 ^ab^	89.5 ± 0.2 ^b^

The parameters of velocity (rapid, slow, and intermediate) refer to total sperm motility. ES = control dilution method, extender to semen; SE = reverse dilution method, semen to extender, RF = sperm rich fraction; TF = total fractions. Different letters (a,b) within a row indicate significant differences between experimental groups (*p* < 0.05).

**Table 4:** Effect of dilution method, ejaculate fraction and vibration on boar spermatozoa conservation after 5 days of refrigeration. Data are shown as the mean ± SEM.

	RF-ES		RF-SE		TF-ES		TF-SE	
Parameter	V	NV	V	NV	V	NV	V	NV
Total Motility (%)	82.9 ± 4.5	86.6 ± 2.7	82.4 ± 3.6	88.3 ± 3.1	86.6 ± 1.7	90.4 ± 3.0	83.9 ± 3.6	86.5 ± 1.1
*Rapid (%)*	44.8 ± 4.9	52.9 ± 4.0	45.3 ± 4.9	55.2 ± 4.8	50.5 ± 3.4	60.3 ± 7.0	48.0 ± 5.0	61.0 ± 7.8
*Intermediate (%)*	19.0 ± 2.4	16.9 ± 2.0	18.1 ± 1.7	16.8 ± 2.3	19.5 ± 3.1	17.3 ± 2.9	18.9 ± 3.3	15.9 ± 3.0
*Slow (%)*	19.0 ± 1.5	16.7 ± 1.4	19.0 ± 1.5	16.2 ± 1.7	16.4 ± 1.8	12.6 ± 1.9	16.9 ± 2.1	14.0 ± 2.3
Progressive Motility (%)	53.9 ± 5.1	59.7 ± 3.5	53.0 ± 4.7	63.7 ± 4.2	61.4 ± 2.9	70.7 ± 5.5	59.0 ± 5.5	69.6 ± 6.2
Non-Progressive Motility (%)	28.9 ± 1.4	26.8 ± 2.3	29.3 ± 1.5	24.6 ± 2.3	25.1 ± 2.2	19.6 ± 2.8	24.8 ± 2.5	21.4 ± 3.8
Normal Morphology (%)	93.3 ± 1.0	92.6 ± 0.8	92.8 ± 1.0	92.8 ± 1.7	93.3 ± 0.5	92.6 ± 1.1	93.0 ± 0.9	93.1 ± 1.1
Viability (%)	94.3 ± 1.2 ^abc^	95.6 ± 0.6 ^c^	92.1 ± 1.3 ^abc^	95.3 ± 1.1 ^bc^	90.1 ± 1.5 ^ab^*	95.3 ± 0.9 ^bc^	89.9 ± 1.2 ^a^**	96.0 ± 1.1 ^c^

The parameters of velocity in italic (rapid, slow, and intermediate) refer to total sperm motility. ES = control dilution method, extender to semen; SE = reverse dilution method, semen to extender; RF = sperm rich fraction; TF = total fractions; V = vibrated; NV = non-vibrated. Different letters (^a,b,c^) within a row indicate significant differences between experimental groups (*p* < 0.05). Asterisk indicates significant differences between vibrated and non-vibrated seminal doses from the same experimental group (* *p* < 0.05; ** *p* < 0.01).

**Table 5:** Effect of dilution method, ejaculate fraction and vibration on boar spermatozoa thermo-resistance after 5 days of refrigeration. Data are shown as the mean ± SEM.

	RF-ES		RF-SE		TF-ES		TF-SE	
Parameter	V	NV	V	NV	V	NV	V	NV
Total Motility (%)	39.5 ± 9.2	37.9 ± 6.6	39.7 ± 7.5	41.1 ± 6.3	33.3 ± 4.3	41.5 ± 4.8	38.3 ± 3.4	40.3 ± 3.0
*Rapid (%)*	15.3 ± 4.7	12.0 ± 3.0	12.2 ± 3.3	14.2 ± 3.5	12.9 ± 2.3	14.5 ± 2.0	12.9 ± 2.2	15.3 ± 2.2
*Intermediate (%)*	7.6 ± 1.9	7.2 ± 1.6	7.6 ± 1.3	8.5 ± 1.4	5.4 ± 1.1	8.0 ± 0.9	7.1 ± 0.7	7.1 ± 1.0
*Slow (%)*	16.5 ± 2.8	18.6 ± 2.8	19.8 ± 3.4	18.3 ± 2.1	14.9 ± 1.2	18.9 ± 3.4	18.3 ± 0.8	17.8 ± 1.2
Progressive Motility (%)	19.3 ± 5.6	16.2 ± 3.7	16.7 ± 3.8	19.1 ± 3.9	16.4 ± 2.9	19.7 ± 2.0	17.4 ± 2.6	19.4 ± 2.1
Non-Progressive Motility (%)	20.2 ± 3.6	21.6 ± 3.3	22.9 ± 3.9	21.9 ± 2.5	16.8 ± 1.6	21.7 ± 3.4	20.9 ± 1.1	20.8 ± 1.6
Normal Morphology (%)	94.5 ± 0.6	94.1 ± 0.7	94.0 ± 0.9	95.0 ± 0.5	93.3 ± 1.1	94.1 ± 0.7	90.8 ± 1.2	93.3 ± 0.7
Viability (%)	91.5 ± 1.0 ^abc^	92.6 ± 1.2 ^bc^	89.6 ± 0.9 ^abc^*	94.3 ± 1.0 ^c^	86.0 ± 2.1 ^ab^	90.0 ± 1.5 ^abc^	85.1 ± 2.7 ^a^	89.5 ± 0.2 ^abc^

The parameters of velocity in italic (rapid, slow, and intermediate) refer to total sperm motility. ES = control dilution method, extender to semen; SE = reverse dilution method, semen to extender; RF = sperm rich fraction; TF = total fractions; V = vibrated; NV = non-vibrated. Different letters (^a,b,c^) within a row indicate significant differences between experimental groups (*p* < 0.05). Asterisk indicates significant differences between vibrated and non-vibrated seminal doses from the same experimental group (*p* < 0.05).

## Data Availability

The original contributions presented in the study are included in the article, and further inquiries can be directed to the corresponding author.

## Supplementary Materials

The following are available online at https://www.mdpi.com/article/10.3390/vetsci8120292/s1. Total motility (%), progressive motility (%), non-progressive motility (%), rapid (%), intermediate (%), slow (%), normal morphology (%), and viability (%) of spermatozoa stored over time (1, 3 and 5 days) in seminal doses containing the rich fraction (RF- grey bars in the graphs) or total fractions (TF- yellow bars in the graphs) of the ejaculate. Seminal doses were prepared pouring the extender over the semen (ES-light grey/yellow bars in the graphs) or pouring the semen over the extender (SE-dark grey/yellow bars in the graph). Data are shown as mean ± SEM.
