# Supplementary material for: Relevance of the Ejaculate Fraction and Dilution Method on Boar Sperm Quality during Processing and Conservation of Seminal Doses
Source: Vet Sci. 2021 Nov 27;8(12):292. doi: 10.3390/vetsci8120292 (PMC8704743; doi:10.3390/vetsci8120292)
Supplement: Supplementary file 1 [file vetsci-08-00292-s001.zip › vetsci-1453312-supplementary.pdf]

veterinary sciences

Supplementary Material

Relevance of the Ejaculate Fraction and Dilution Method on Boar Sperm Quality during Processing and Conservation of Seminal Doses

Blanca Sebastián-Abad, Pedro José Llamas-López and Francisco Alberto García-Vázquez

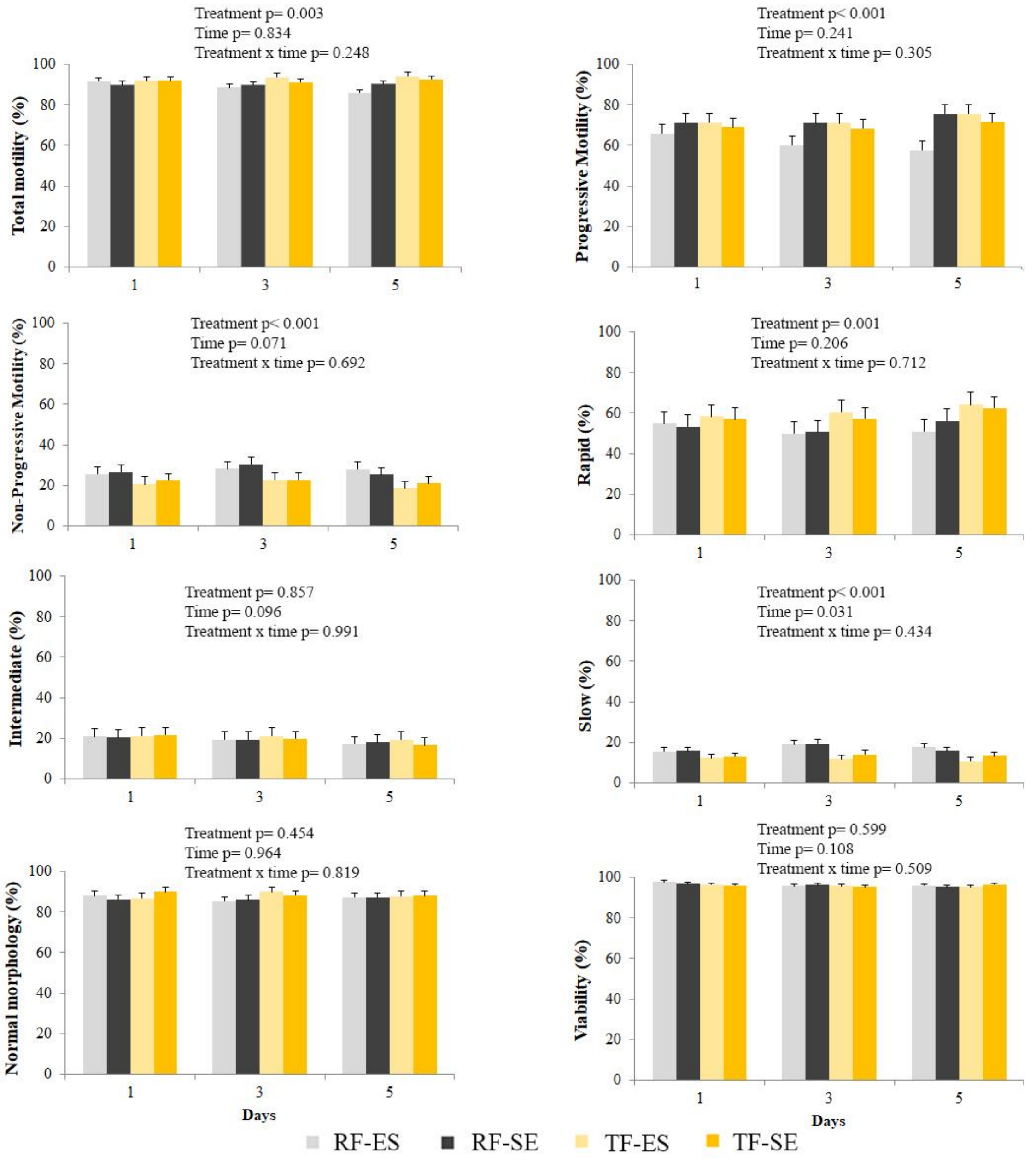

Supplementary file. Total motility (%), progressive motility (%), non-progressive motility (%), rapid (%), intermediate (%), slow (%), normal morphology (%), and viability (%) of spermatozoa stored over time (1, 3 and 5 days) in seminal doses containing the rich fraction (RF- grey bars in the graphs) or total fractions (TF- yellow bars in the graphs) of the ejaculate. Seminal doses were

prepared pouring the extender over the semen (ES-light grey/yellow bars in the graphs) or pouring the semen over the extender (SE-dark grey/yellow bars in the graph). Data are shown as mean ± SEM.
